# Evaluation of intravenous lidocaine in head and neck cancer surgery: study protocol for a randomized controlled trial

**DOI:** 10.1186/s13063-019-3303-x

**Published:** 2019-04-15

**Authors:** Edris Omar, Grégoire Wallon, Christian Bauer, Grégory Axiotis, Cécile Bouix, Jean-Luc Soubirou, Frédéric Aubrun

**Affiliations:** 1 Department of Anesthesiology and Critical Care, Croix-Rousse Hospital, Hospices Civils de Lyon, Lyon, France; 2 Department of Anesthesiology and Critical Care, Centre Léon Bérard, Lyon, France; 3 Clinical Research Center, Croix-Rousse Hospital, Hospices Civils de Lyon, Lyon, France

## Abstract

**Background:**

Pain after major head and neck cancer surgery is underestimated and has both nociceptive and neuropathic characteristics. Extended resection, flap coverage, nerve lesions, inflammation, and high-dose opioid administration can also lead to hyperalgesia and chronic postoperative pain. Opioids are frequently associated with adverse events such as dizziness, drowsiness, nausea and vomiting, or constipation disturbing postoperative recovery and extending the length of hospital stay. Patients eligible for major head and neck cancer surgery cannot benefit from full multimodal pain management with locoregional anesthesia. Intravenous lidocaine, investigated in several studies, has been found to decrease acute pain and morphine consumption. Some data suggest also that it can prevent chronic postsurgical pain. Evidence supporting its use varies between surgical procedures, and there is no published study regarding systemic lidocaine administration in major head and neck cancer surgery. We hypothesized that intravenous lidocaine infused in the perioperative period would lead to opioid sparing and chronic postsurgical pain reduction.

**Methods/design:**

A total of 128 patients undergoing major head and neck surgery will be included in this prospective two-center, double-blind, randomized controlled trial. Patients will be randomly assigned to lidocaine or placebo treatment. After induction of general anesthesia, an intravenous lidocaine bolus will be administered (1.5 mg.kg^− 1^), followed by a continuous infusion (2 mg.kg^− 1^.h^− 1^) which will be reduced in the postanesthesia care unit (1 mg.kg^− 1^.h^− 1^). The primary outcome measure is morphine consumption 48 h after surgery. The secondary outcomes include intraoperative remifentanil consumption, morphine consumption 24 h after surgery, and chronic postsurgical pain that will be assessed 3–6 months after surgery.

**Discussion:**

Recent evidence suggests that intravenous lidocaine can lead to opioid sparing and chronic postsurgical pain reduction for certain types of surgery. This is the first trial to prospectively investigate the efficacy and safety of intravenous lidocaine in major head and neck cancer surgery.

**Trial registration:**

ClinicalTrials.gov, NCT02894710. Registered on 11 August 2016.

**Electronic supplementary material:**

The online version of this article (10.1186/s13063-019-3303-x) contains supplementary material, which is available to authorized users.

## Background

Intravenous lidocaine has spurred an increased interest in the use of alternative perioperative nonopioid analgesia after major surgery [1]. Morphine has long been considered the gold standard to relieve pain after most surgical procedures. However, opioid-related adverse events, such as postoperative nausea and vomiting (PONV), constipation, itching, sedation, drowsiness, dizziness, and respiratory depression, may disturb postoperative recovery and extend the length of hospital stay. Furthermore, neuronal activation and central neuroplasticity generated by intense nociceptive stimulation and high-dose opioid administration can result in hyperalgesia and chronic postsurgical pain (CPSP) [2]. Thus, opioid sparing by an antihyperalgesic compound such as lidocaine could prevent CPSP [3–5]. Systemic lidocaine, used initially as an antiarrhythmic drug, has a very short half-life and a favorable safety profile for systemic administration [6]. Through its analgesic and anti-inflammatory activities, systemic lidocaine enhances postoperative recovery by opioid sparing and by reducing immune alterations [7]. A recent meta-analysis found that the efficacy of perioperative intravenous lidocaine for postoperative pain varies between surgical procedures [8]. Indeed, opioid sparing was observed after lidocaine infusion in open abdominal [9, 10] and laparoscopic [11, 12] procedures, thyroid surgery [13], and cardiac [14], thoracic [15], and major spine [16] procedures. Moreover, data suggest a reduction of CPSP development in breast surgery [4, 17]. The published data being inconsistent, the efficacy of lidocaine infusion has to be assessed for each surgical procedure. There is no published study that has evaluated intravenous lidocaine in major head and neck cancer surgery. In these procedures, acute and CPSP are major and underestimated issues [18–20]. Patients are not eligible for a full multimodal pain management with locoregional anesthesia. The head and neck region is widely innervated. Erosive tumor, inflammation, and extended surgical resections can lead to acute postoperative pain that has various characteristics (nociceptive, neuropathic, and psychological). Insufficient pain relief, nerve lesions, and high opioid consumption can result in hyperalgesia and chronicization of the pain. We hypothesized that perioperative intravenous lidocaine would lead to opioid sparing and CPSP reduction, and therefore designed a study to investigate this, the protocol of which is reported herein.

## Methods/design

### Study design

**Fig. 1:**
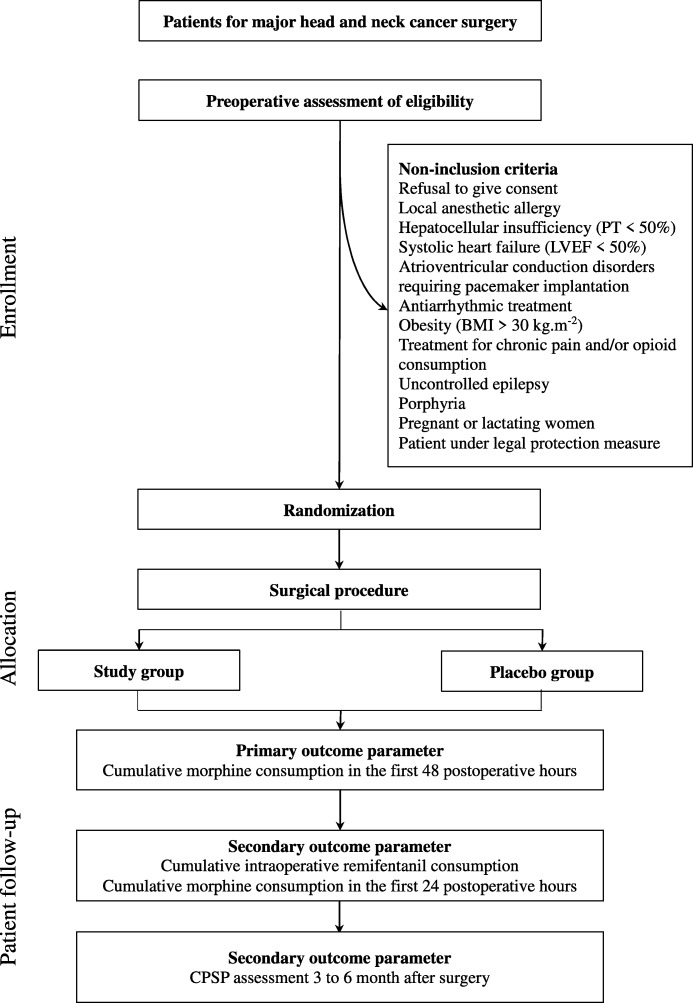
Flow chart of the study. BMI body mass index, CPSP chronic post-surgical pain, LVEF left ventricular ejection fraction, PT prothrombin time

**Fig. 2:**
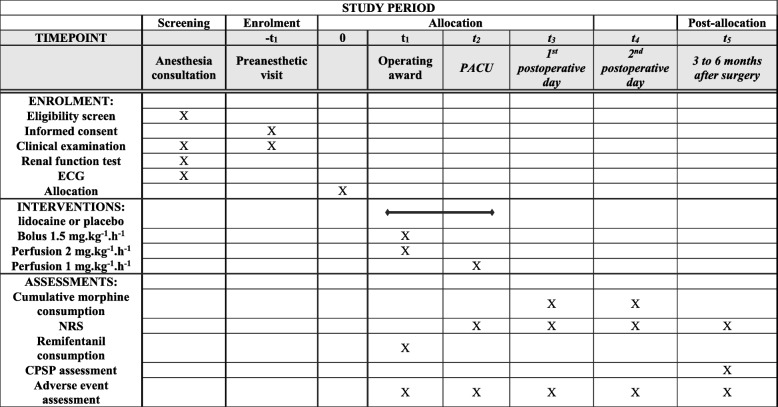
Schedule of study assessments and evaluations. CPSP chronic postsurgical pain, ECG electrocardiogram, NRS numeric rating scale, PACU postanesthesia care unit

This study is a two-center, double-blind, randomized controlled prospective trial. It was approved by the local ethics committee (CPP Sud-Est II) on July 6, 2016, and by the French medicines agency (Agence nationale de sécurité du médicament et des produits de santé (ANSM)) on July 7, 2016. The trial is registered on EUDRA-CT (2015-005799-90) and on clinicaltrials.gov (NCT02894710). The trial design is illustrated in Figs. 1 and 2.

### Population

All patients scheduled for elective major head and neck cancer surgery at the Croix-Rousse Hospital (Hospices Civils de Lyon, France) and the Centre Léon Bérard (Lyon, France) will be included after written informed consent is obtained.

### Inclusion criteria

Participants meeting the following criteria will be included: patients undergoing major head and neck cancer surgery (total or partial laryngectomy, crico-hyoido-epiglotto-pexy, oropharyngectomy with or without mandibulotomy, partial pharyngectomy, intraoral extended resection, extended pelvectomy, pelvi-glossectomy with or without pectoralis major flap or free flap with or without cervical lymphadenectomy) and patients receiving standardized morphine patient-controlled analgesia (PCA) in the postoperative period.

### Non-inclusion criteria

Participants meeting one or more of the following criteria will be excluded: refusal to give consent; hypersensitivity to amide local anesthetics; hepatocellular insufficiency (international normalized ratio > 1.6) or cirrhosis; systolic heart failure (left ventricular ejection fraction < 50%); atrioventricular conduction disorders requiring permanent electrosystolic stimulation with pacemaker not yet implemented; treatment with antiarrhythmic or beta-blockers classified by Vaughan Williams; body mass index > 30 kg.m^− 2^; patient already treated for chronic pain with a level 3 analgesic or for neuropathic pain; epilepsy not controlled by treatment; acute porphyria; fluid overload; hypersensitivity to any component of 5% glucose solution; pregnant or lactating women; and patient under legal protection measure.

### Randomization

Patients will be allocated by randomized complete block design, stratified by center, to one of the two groups that will receive either intravenous lidocaine (study group) or placebo (placebo group) during the intraoperative and early postoperative period. The randomization will be processed by the investigator after the preoperative assessment, using a computer-generated list. Allocation concealment will be transmitted to the data manager and to each pharmacy department. Allocation will be ensured by enclosing the treatment unit in sealed, opaque, sequentially numbered envelopes prepared by the pharmacy department, which will be opened only upon arrival of the patient in the operation room by a nurse who will prepare the syringes. The nurse will prepare the treatment unit away from the operating room in order to keep the study participants blinded from the allocation. Preparations will consist of two 50-ml syringes for continuous infusion by syringe pump. Postoperative outcomes will be assessed by research personnel who will remain blinded to the type of intervention throughout the study.

### Intervention plan

For all patients, a standardized general anesthesia and postoperative care protocol will be followed in each center. Anesthesia is performed by the staff anesthesiologist, who does not participate in postoperative evaluation and who is blinded to the patient’s allocation.

### Induction and maintenance of anesthesia

After preoxygenation, anesthesia will be induced by a bolus injection of propofol and an intravenous infusion of remifentanil (Minto model for remifentanil target-controlled infusion adapted to arterial pressure and heart rate variations). Tracheal intubation may be facilitated with a neuromuscular blocking agent according to the clinicians’ decision. Patients will be mechanically ventilated with a mixture of air, oxygen, and sevoflurane. It may be noted that antihyperalgesic agents, such as nitric oxide, ketamine, or nonsteroidal anti-inflammatory drugs, are not allowed during the protocol [21–23]. In all patients, standard monitoring will be applied, including electrocardiogram, pulse oximetry, capnography, and temperature measurements.

### Interventional treatment

In the study group, at the end of the induction of general anesthesia, a bolus of 1.5 mg.kg^− 1^ of intravenous lidocaine will be administered, followed by a continuous infusion of intravenous lidocaine at 2 mg.kg^− 1^.h^− 1^. The lidocaine infusion will be reduced to 1 mg.kg^− 1^.h^− 1^ in the postanesthesia care unit (PACU) and will be discontinued at the time of discharge. The placebo group will receive, in the perioperative period, a placebo infusion with 5% glucose solution at a rate comparable to that of the lidocaine infusion in the study group.

### Postoperative analgesia

Irrespective of group allocation, all patients will receive a combination of intravenous analgesics 30 min before the end of surgery: acetaminophen 1 g and morphine 0.15 mg.kg^− 1^. The patients will be transferred to the PACU for continuous monitoring of vital signs. The Aldrete score will be recorded before leaving the PACU [24]. The level of pain will be assessed using a numeric rating scale (NRS: 0 = no pain, 10 = worst pain you can imagine). As soon as the NRS score exceeds 3, patients will receive a 2 mg intravenous bolus dose of morphine every 10 min [25]. Morphine titration is performed until pain relief. Afterward, the morphine PCA pump will be programmed in an on-demand-only mode without a basal rate, allowing a bolus injection of 1 mg every 7 min without a maximum programmed dose. Patients will be discharged from the PACU only once the Aldrete score is 9, and once there is no evidence of pain and/or PONV.

### Postoperative management

Patients will receive intravenous analgesia with acetaminophen 1 g every 6 h and morphine PCA during at least the first 48 h after the procedure. The fluid balance, enteral nutrition, antibiotic use, and thromboembolic prevention will be at the clinicians’ decision.

### Follow-up visits

The first follow-up visit will be processed in the PACU for assessment of morphine consumption and adverse effects. Two other visits are planned at day 1 and day 2 of the early postoperative period for morphine consumption assessment. The last visit will be 3–6 months after surgery to evaluate CPSP with the NRS and the shortened version of the “Questionnaire Douleur Saint-Antoine” (QDSA), which is the French adaptation of the McGill Pain Questionnaire (MPQ) [26]. Each new analgesic or anti-inflammatory drug or any relevant therapy (such as chemotherapy, radiotherapy) will be noted.

### Primary outcome

The primary outcome is the cumulative administered morphine consumption in the first 48 postoperative hours.

### Secondary outcome

Secondary outcomes include: cumulative remifentanil consumption during surgery; cumulative morphine consumption in the first 24 postoperative hours; assessment of CPSP 3–6 months after surgery using the shortened version of the QDSA; and occurrence of adverse events.

### Assessment of safety

The interventional treatment will be administered to patients with standard hemodynamic monitoring in the setting of a fully equipped operation theater. This enables immediate detection and treatment of adverse events. Administration of study drug will be immediately stopped in cases when the study participant shows a relevant deterioration. Also, after leaving the operation room, all patients will be closely monitored for the occurrence of adverse events, first in the PACU and later in the surgical ward. Moreover, the inclusion of each patient into the study will be noted in the electronic hospital information system and is hence visible to all physicians and nurses involved in the care of the patient. This facilitates reporting of severe adverse events to the principal investigator. The principal investigator will report suspected unexpected serious adverse reactions to the national health authorities.

### Data analysis

Data will be analyzed according to the intention-to-treat principle. Unblinding is planned at the end of the study, after a database freeze. A descriptive analysis will first be performed. The data will be presented as counts and percentages for qualitative data, as means and standard deviation for normally distributed quantitative data, or as medians and interquartile range for non-normally distributed variables. The analysis of the main outcome (cumulative morphine consumption in the first 48 postoperative hours) will be performed using Student’s *t* test. A multivariate linear regression analysis will also be performed to identify factors independently associated with morphine consumption 48 h after surgery. The factors studied will be the type of surgery, tumor site, stage of cancer, duration of the surgery, NRS score upon arrival in the PACU, use of preoperative analgesic, and use of high-dose intraoperative remifentanil. During surgery, intraoperative remifentanil consumption as well as morphine consumption in the 24 postoperative hours will be compared between the two groups using Student’s *t* test. The incidence of postoperative chronic pain will be compared between the two groups and the factors potentially associated with the occurrence of chronic pain will be analyzed by logistic regression. Subgroup analysis will be performed to compare patients with and without major pectoralis flap or free flap. The statistical analyses will be carried out under the responsibility of the methodologist of the clinical research center of the Croix-Rousse Hospital. All analyses will be conducted using SPSS software version 19.0 (IBM Corp., Armonk, NY, USA) and R software (R Development Core Team, Vienna, Austria). A value of *p* < 0.05 will be considered statistically significant.

### Sample size calculation

The study has been powered to detect a reduction of 20% of morphine consumption outcome between the study and placebo groups. Data from the head and neck surgery department of the Croix-Rousse Hospital permitted an estimation, in the absence of lidocaine administration, of a mean morphine consumption of 43 mg at day 2 (standard deviation 17 mg). With a statistical power of 0.80, 114 patients (57 per group) will be needed to demonstrate such a difference (Student’s *t* test). To compensate for a possible dropout of 10% of patients, it is planned to include a total of 128 patients (64 per group).

### Protocol amendment

In order to extend inclusions, the protocol was amended (October 2016) to also include women, as well as patients undergoing total laryngectomy and interventions completed by free flap coverage.

## Discussion

### Benefits

Recently, intravenous lidocaine has been shown to effectively control postoperative pain according to the surgical procedure. Pain relief is reported to persist for 48 h after infusion [8]. There are no published data regarding systemic lidocaine in major head and neck cancer surgery. We hypothesized that perioperative intravenous lidocaine would lead to opioid sparing and CPSP reduction.

The benefit of lidocaine for pain relief is not likely to be due to sodium channel blockade regarding the perioperative relatively low blood concentrations [8]. It may therefore be related to a priming blockade of granulocytes, which could limit the exaggerated release of cytokines and reactive oxygen species [27], even effective at very low concentration [28].

Lidocaine is widely used intravenously in the perioperative period for abdominal and urological procedures and could lead to opioid sparing, through analgesic and anti-inflammatory properties [8]. Moreover, by an antihyperalgesic effect, systemic lidocaine could prevent CPSP [3–5]. Assessment of hyperalgesia and CPSP is permitted as we tried to limit the use of antihyperalgesic drugs (such as nitric oxide, ketamine, and nonsteroidal anti-inflammatory drugs) [21–23].

Moreover, surgery-induced stress and anesthetic-induced and opioid-induced immunosuppression during the perioperative period may play a critical role in the establishment and growth of metastatic lesions. The role of anesthetic-induced immunosuppression in the promotion of cancer recurrence is suggested by currently available preclinical studies [29] (Additional file 1).

### Risks

Medical care of the included patients, in terms of anesthesia, surgery, and postoperative care, will not differ from routine practice. Risks are mainly related to the systemic toxicity of local anesthetics; an overdose may affect the central nervous system (e.g. drowsiness, confusion, euphoria, double vision, seizures), the cardiovascular system (e.g. hypotension, bradycardia, arrhythmias, cardiac arrest), the respiratory system (e.g. tachypnea, apnea), or the blood system (e.g. methemoglobinemia). However, the doses used in the present study have been repeatedly demonstrated to be safe and to result in plasma concentrations that are far below the toxic level (5 μg.ml^− 1^) [8]. As a safety measure, patients with cardiac systolic dysfunction or impaired lidocaine metabolism due to liver dysfunction or cirrhosis are excluded from the study [6]. Moreover, patients are continuously monitored by electrocardiography during the administration of lidocaine. If intoxication is suspected, the patient will receive an infusion of lipid emulsion after standard resuscitative measures according to local protocols based on international guidelines [30].

### Limits

The study does, however, have several limitations. One limitation is the recruitment of patients undergoing various types of procedure. Surgical techniques will be obviously different according to each procedure, and therefore we have planned subgroup analyses (with or without major pectoralis flap or free flap). Another limitation is that, for organizational issues, treatment units have to be prepared by a PACU nurse who then brings the syringe to the operating theater but who will not be involved in the care of the study patient. Although this aims to guarantee blinding, as the nurse and the medical team treating the patient will be in the same department then a bias due to unblinding cannot be ruled out. Furthermore, if use of postoperative corticosteroids was expected by the surgical team, the patient was not enrolled. However, if corticosteroids are infused in the perioperative period because of a potential upper airway edema, the patient will not be excluded; analyses conducted according to the intention-to-treat principle will be used for analysis, and sub-group analysis will be considered according to each group’s workforce.

### Trial status

Patient recruitment started in December 2016. The predicted study completion date is June 2019.

## Additional file

Additional file 1: SPIRIT 2013 checklist: recommended items to address in a clinical trial protocol and related documents. (DOC 122 kb)

## Abbreviations

CPSP: Chronic postsurgical pain
MPQ: McGill Pain Questionnaire
NRS: Numeric rating scale
PACU: Postanesthesia care unit
PCA: Patient-controlled analgesia
PONV: Postoperative nausea and vomiting
QDSA: Questionnaire de Saint-Antoine (French version of MPQ)

## References

[1] Aubrun F, Nouette Gaulain K, Fletcher D, Belbachir A, Beloeil H, Carles M (2016). Réactualisation de la recommandation sur la douleur postopératoire. Anesth Reanim.

[2] Guignard B, Bossard AE, Coste C, Sessler DI, Lebrault C, Alfonsi P (2000). Acute opioid tolerance: intraoperative remifentanil increases postoperative pain and morphine requirement. Anesthesiology.

[3] Fletcher D, Martinez V (2014). Opioid-induced hyperalgesia in patients after surgery: a systematic review and a meta-analysis. Br J Anaesth.

[4] Grigoras A, Lee P, Sattar F, Shorten G (2012). Perioperative intravenous lidocaine decreases the incidence of persistent pain after breast surgery. Clin J Pain.

[5] Pogatzki-Zahn E, Segelcke D, Zahn P. Mechanisms of acute and chronic pain after surgery: update from findings in experimental animal models. Curr Opin Anaesthesiol. 2018;31:575-85. 10.1097/ACO.0000000000000646 30028733

[6] Collinsworth KA, Kalman SM, Harrison DC (1974). The clinical pharmacology of lidocaine as an antiarrhythymic drug. Circulation.

[7] Yardeni IZ, Beilin B, Mayburd E, Levinson Y, Bessler H (2009). The effect of perioperative intravenous lidocaine on postoperative pain and immune function. Anesth Analg.

[8] Weibel S, Jelting Y, Pace NL, Helf A, Eberhart LH, Hahnenkamp K (2018). Continuous intravenous perioperative lidocaine infusion for postoperative pain and recovery in adults. Cochrane Database Syst Rev.

[9] Kuo CP, Jao SW, Chen KM, Wong CS, Yeh CC, Sheen MJ (2006). Comparison of the effects of thoracic epidural analgesia and i.v. infusion with lidocaine on cytokine response, postoperative pain and bowel function in patients undergoing colonic surgery. Br J Anaesth.

[10] Swenson BR, Gottschalk A, Wells LT, Rowlingson JC, Thompson PW, Barclay M (2010). Intravenous lidocaine is as effective as epidural bupivacaine in reducing ileus duration, hospital stay, and pain after open colon resection: a randomized clinical trial. Reg Anesth Pain Med.

[11] Kaba A, Laurent SR, Detroz BJ, Sessler DI, Durieux ME, Lamy ML (2007). Intravenous lidocaine infusion facilitates acute rehabilitation after laparoscopic colectomy. Anesthesiology.

[12] Lauwick S, Kim DJ, Mistraletti G, Carli F (2009). Functional walking capacity as an outcome measure of laparoscopic prostatectomy: the effect of lidocaine infusion. Br J Anaesth.

[13] Choi GJ, Kang H, Ahn EJ, Oh JI, Baek CW, Jung YH (2016). Clinical efficacy of intravenous lidocaine for thyroidectomy: a prospective, randomized, double-blind, placebo-controlled trial. World J Surg.

[14] Wang D, Wu X, Li J, Xiao F, Liu X, Meng M (2002). The effect of lidocaine on early postoperative cognitive dysfunction after coronary artery bypass surgery. Anesth Analg.

[15] Cui W, Li Y, Li S, Wang R, Li J (2010). Systemic administration of lidocaine reduces morphine requirements and postoperative pain of patients undergoing thoracic surgery after propofol-remifentanil-based anaesthesia. Eur J Anaesthesiol.

[16] Farag E, Ghobrial M, Sessler DI, Dalton JE, Liu J, Lee JH (2013). Effect of perioperative intravenous lidocaine administration on pain, opioid consumption, and quality of life after complex spine surgery. Anesthesiology.

[17] Terkawi AS, Sharma S, Durieux ME, Thammishetti S, Brenin D, Tiouririne M (2015). Perioperative lidocaine infusion reduces the incidence of post-mastectomy chronic pain: a double-blind, placebo-controlled randomized trial. Pain Physician.

[18] Sommer M, Geurts JWJM, Stessel B, Kessels AGH, Peters ML, Patijn J (2009). Prevalence and predictors of postoperative pain after ear, nose, and throat surgery. Arch Otolaryngol Head Neck Surg.

[19] Burton AW, Fanciullo GJ, Beasley RD, Fisch MJ (2007). Chronic pain in the cancer survivor: a new frontier. Pain Med.

[20] Pang J, Tringale KR, Tapia VJ, Moss WJ, May ME, Furnish T (2017). Chronic opioid use following surgery for oral cavity cancer. JAMA Otolaryngol Head Neck Surg.

[21] Levy D, Zochodne DW (2004). NO pain: potential roles of nitric oxide in neuropathic pain. Pain Pract.

[22] Bell RF, Dahl JB, Moore RA, Kalso E. Perioperative ketamine for acute postoperative pain. Cochrane Database Syst Rev. 2006;(1):CD004603. 10.1002/14651858.CD004603.pub2 16437490

[23] Burian M, Geisslinger G (2005). COX-dependent mechanisms involved in the antinociceptive action of NSAIDs at central and peripheral sites. Pharmacol Ther.

[24] Aldrete JA (1995). The post-anesthesia recovery score revisited. J Clin Anesth.

[25] Aubrun F, Valade N, Riou B (2004). Intravenous morphine titration. Ann Fr Anesth Reanim.

[26] Melzack R (1975). The McGill Pain Questionnaire: major properties and scoring methods. Pain.

[27] Leliefeld PHC, Wessels CM, Leenen LPH, Koenderman L, Pillay J (2016). The role of neutrophils in immune dysfunction during severe inflammation. Crit Care.

[28] Nishina K, Mikawa K, Takao Y, Shiga M, Maekawa N, Obara H (1998). Intravenous lidocaine attenuates acute lung injury induced by hydrochloric acid aspiration in rabbits. Anesthesiology.

[29] Kim R (2018). Effects of surgery and anesthetic choice on immunosuppression and cancer recurrence. J Transl Med.

[30] Neal JM, Woodward CM, Harrison TK (2018). The American Society of Regional Anesthesia and Pain Medicine Checklist for Managing Local Anesthetic Systemic Toxicity: 2017 Version. Reg Anesth Pain Med.

